# Advancements in Fibrinogen Replacement for Major Bleeding: Insights From Case Series

**DOI:** 10.7759/cureus.94480

**Published:** 2025-10-13

**Authors:** David S Shapiro, Daniel Katz, John J Kowalczyk, Grace Lim, Roman Dudaryk, Tjorvi Perry

**Affiliations:** 1 Department of Surgery, University of Connecticut School of Medicine, Farmington, USA; 2 Department of Anesthesiology, The Mount Sinai Hospital, New York, USA; 3 Department of Anesthesiology, Perioperative and Pain Medicine, Brigham and Women's Hospital, Harvard Medical School, Boston, USA; 4 Department of Anesthesiology, University of Pittsburgh Medical Center, Pittsburgh, USA; 5 Department of Anesthesiology, University of Miami, Coral Gables, USA; 6 Department of Anesthesiology, University of Minnesota, Minneapolis, USA

**Keywords:** anesthesiology, bleeding, bleeding and blood products, cardiac surgery, fibrinogen, obstetrical hemorrhage, resuscitation, traumatic hemorrhage

## Abstract

This case series explores the role of fibrinogen replacement in managing major bleeding across cardiac, obstetric, and trauma contexts. Acquired fibrinogen deficiency (AFD) frequently complicates hemostasis during major bleeding by impairing clot stability. We examine three cases where fibrinogen is repleted with fibrinogen concentrate (FC) or pathogen-reduced cryoprecipitate, rather than cryoprecipitate, to manage perioperative bleeding in cardiac surgery, postpartum hemorrhage, and trauma-induced coagulopathy. These cases underscore the need for timely fibrinogen replacement in critical bleeding situations, highlighting potential clinical benefits of rapid repletion of fibrinogen, and, in addition, we review ideal treatment options based upon viscoelastic testing (VET).

## Introduction

Patients experiencing major bleeding often face life-threatening consequences in cardiac, obstetric, and trauma surgeries. One in 10 cardiac surgeries is associated with severe perioperative bleeding [1]. Postpartum hemorrhage (PPH) is the leading direct cause of maternal death worldwide, and hemorrhage is the most frequent cause of death in victims of severe trauma, with ~30% of patients developing trauma-induced coagulopathy [2,3]. Acquired fibrinogen deficiency (AFD) is common in major hemorrhage across all scenarios, and fibrinogen replacement plays a pivotal role in correcting coagulopathy [4].

Fibrinogen, a plasma glycoprotein, is essential for clot stability and hemostasis [4]. Fibrinogen, consumed during coagulation, is the first coagulation factor to reach critical levels during major bleeding episodes [5]. The etiology of AFD in major bleeding can vary [6]. In cardiac surgeries with cardiopulmonary bypass (CPB), contact with the CPB circuit exacerbates consumption of factors, while hemodilution may result from CPB priming and intravenous fluid administration [4]. In the obstetric setting, in addition to hemodilution, blood factor consumption can be rapidly accelerated, particularly in placental abruption, amniotic fluid embolism, and placenta accreta [4]. AFD develops in severely injured patients with a combination of increased consumption, hyperfibrinolysis, and the use of resuscitation modalities that lack appropriate concentrations of fibrinogen.

The specific threshold of AFD varies by clinical scenario but is often defined as below 150 mg/dL [7], and levels below 200 mg/dL have been shown to predict massive blood loss and the need for transfusion [8]. In the setting of PPH in particular, where the baseline fibrinogen level is higher, fibrinogen levels below 200 mg/dL have a 100% positive predictive value of progressing to severe hemorrhage requiring transfusion [9].

Anesthesiologists and surgeons have numerous tools for managing hemostasis in these circumstances, including whole blood transfusion, blood component therapy (fresh frozen plasma (FFP), cryoprecipitate, pathogen-reduced cryoprecipitate, platelets), blood derivatives (fibrinogen concentrate (FC), prothrombin concentrates, coagulation factor concentrates in single or multiple factor agents), and other therapeutic options (tranexamic acid, aminocaproic acid, desmopressin) [10,11]. The potential advantages of FC compared to cryoprecipitate are discussed in the next section [12]. In July 2024, FC became the first pharmacological product to be approved by the US Food and Drug Administration for AFD in bleeding patients. In this case series, we consider approaches to managing the bleeding patient in the context of AFD. Health Insurance Portability and Accountability Act (HIPAA) authorization was obtained for all cases.

## Case presentation

A synopsis of all TEG results and reference ranges is presented in Table 1.

**Table 1:** Case-specific testing results R-time: time to start of clot formation, measuring all coagulation factors; K-time: time until clot reaches fixed strength, measuring fibrinogen; alpha angle: speed of fibrin accumulation, measuring fibrinogen; MA: maximum vertical amplitude of TEG^TM^ measure, determining platelet function; Ly30: percentage of amplitude reduction 30 minutes after MA, measuring fibrinolysis. Reference ranges based upon Haemoscope TEG 5000 Thromboelastograph Hemostasis System User Manual [12]. * Some parameters and normal levels are reported based upon particular device manufacturers and types; review your device instructions for use.

TEG^TM^ parameters*	Reference Ranges*		Units	Interpretation	Actions Considered
	Standard TEG	Rapid-TEG			
R-time	(5-10)	(0.3-1.1)	minutes	Reaction time - indicates the time until initial clot formation. Suggests the efficiency and rate-limiting steps in the coagulation cascade.	If prolonged, consider providing coagulation factor deficiencies, including fresh frozen plasma, factor concentrates, or preparations.
K-time	(1-3)	(0.9-2)	minutes	Clot formation time - time until the clot, once initiated, reaches fixed strength; starts when R-time ends and reflects the rate of clot amplification and stabilization.	If prolonged, consider cryoprecipitate or fibrinogen supplementation.
α-angle (alpha)	(53-72)	(64-80)	degrees	Reflects the crosslinking and strengthening rate of the clot.	If higher than range, monitor for hypercoagulable states; if lower than range, administer cryoprecipitate or fibrinogen concentrate.
Maximum amplitude (MA)	(50-70)	(52-71)	mm	Measures platelet function and the strength of the fibrin clot.	If higher than range, consider antiplatelet therapy; if lower, administer platelet concentrates.
Lysis at 30 minutes (Ly30)	(0-8)	(0-2.2)	% lysis	Measures fibrinolysis or breakdown of the clot.	If elevated, administer antifibrinolytic agents like tranexamic acid; if normal or reduced, no actions are required.

Cardiac

**Figure 1:**
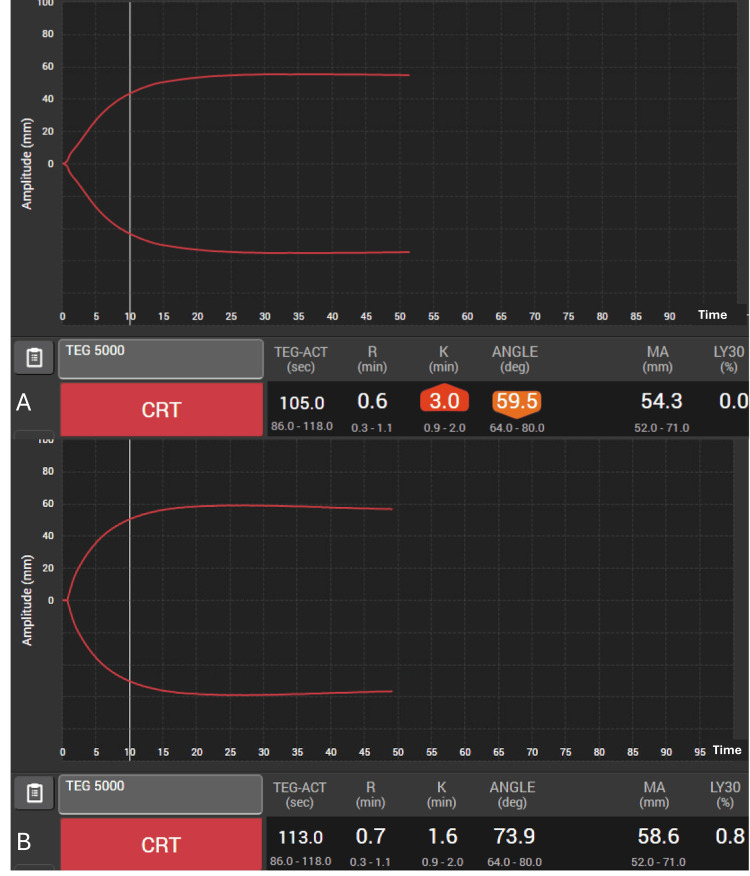
(A) Point-of-care R-TEG showing prolonged K time (3 min) and reduced alpha angle (59.5°), consistent with AFD-related coagulopathy performed pre-treatment. (B) Point-of-care R-TEG results showing K time and alpha angle normalized after fibrinogen concentrate (fibryga®, Fibrinogen (Human), Octapharma, Paramus, NJ, USA) administration. R: reaction time; MA: maximum amplitude; LY30: lysis at 30 minutes; CK: citrated kaolin; CRT: citrated rapid TEG assay; AFD: acquired fibrinogen deficiency

A 69-year-old, 90 kg male patient presented for a surgical aortic valve and root replacement for severe aortic valve stenosis. He had a history of coronary artery disease and underwent coronary artery bypass graft surgery 10 years prior. The patient was cooled to 28 degrees Celsius for the operation. After 210 minutes on CPB and subsequent rewarming, the patient was ready to wean from CPB. This patient had several risk factors for increased microvascular bleeding, including reoperation, a relatively long CPB time, and intraoperative cooling. Following successful weaning from CPB, diffuse bleeding was noted throughout the surgical field, including the pericardium, pericardial fat, and sternum. Post-CPB labs, including coagulation studies, were pending. A point-of-care VET (Thromboelastography or TEG^TM^, Haemonetics, Boston, MA, USA) demonstrated a prolonged K time of 3 minutes (normal 1-3 minutes) and a decreased alpha angle of 59.5 degrees (normal 64-80), suggesting that AFD was the most likely etiology for the coagulopathy (Figure 1).

While awaiting fibrinogen levels, a dose of 70 mg/kg body weight of fibrinogen was prepared as FC (fibryga®, Fibrinogen (Human), Octapharma, Paramus, NJ, USA) per the package insert and standing protocol, and immediately administered as a starting dose. Anticipating platelet sequestration from cooling and despite adequate rewarming, two units of apheresis platelets were also administered. Following these therapies, the surgeons noted a clot in the surgical field and repeated TEG^TM^, which normalized. The pre-CPB fibrinogen level of 92 mg/dL had increased to 140 mg/dL after FC administration, and as the coagulopathy improved and bleeding subsided despite a fibrinogen level less than 150 mg/dL, no additional FC was administered. The chest was closed, and the patient was transferred to intensive care for continued management.

Obstetric

**Figure 2:**
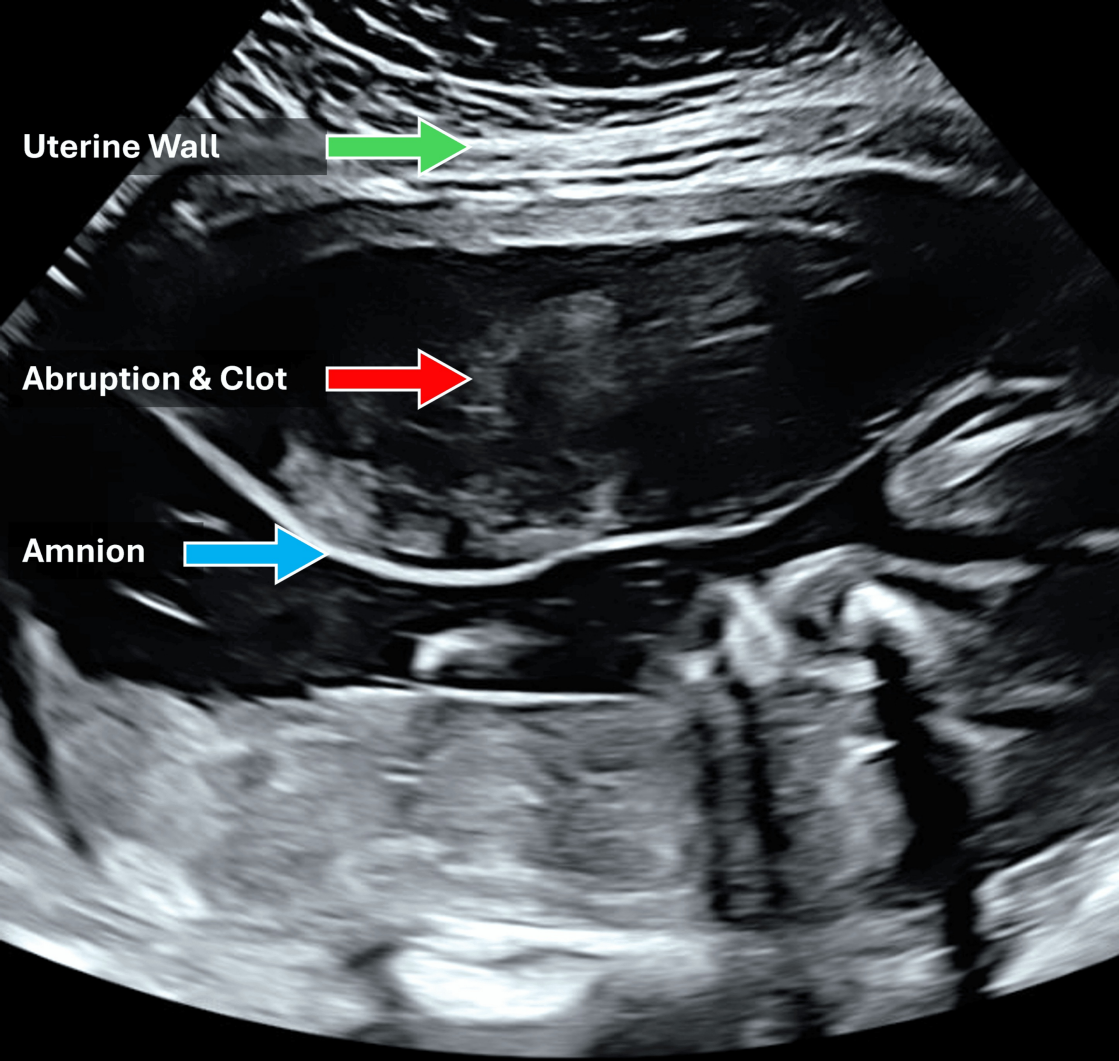
Transabdominal sonogram demonstrating acute placental abruption. An isoechoic layering appears to extend across the width of the sonogram field with a defined area of clot between the wall and amniotic sac.

A 29-year-old, 71 kg, gravida 4, para 2 pregnant patient at 32 weeks and four days gestation with a history of two large uterine fibroids and PPH after her last repeat cesarean delivery presented to labor and delivery from the antepartum assessment clinic with her third episode of vaginal bleeding. The patient had a presumed abruption based on bleeding history and ultrasound results (Figure 2).

The patient was experiencing painful contractions with bright red blood present on exam, with a reassuring category I fetal heart rate (FHR) tracing. Initial labs showed a hematocrit of 31.4%, platelet count of 187 K/μL, international normalized ratio (INR) of 1.0, and fibrinogen of 559 mg/dL.

**Figure 3:**
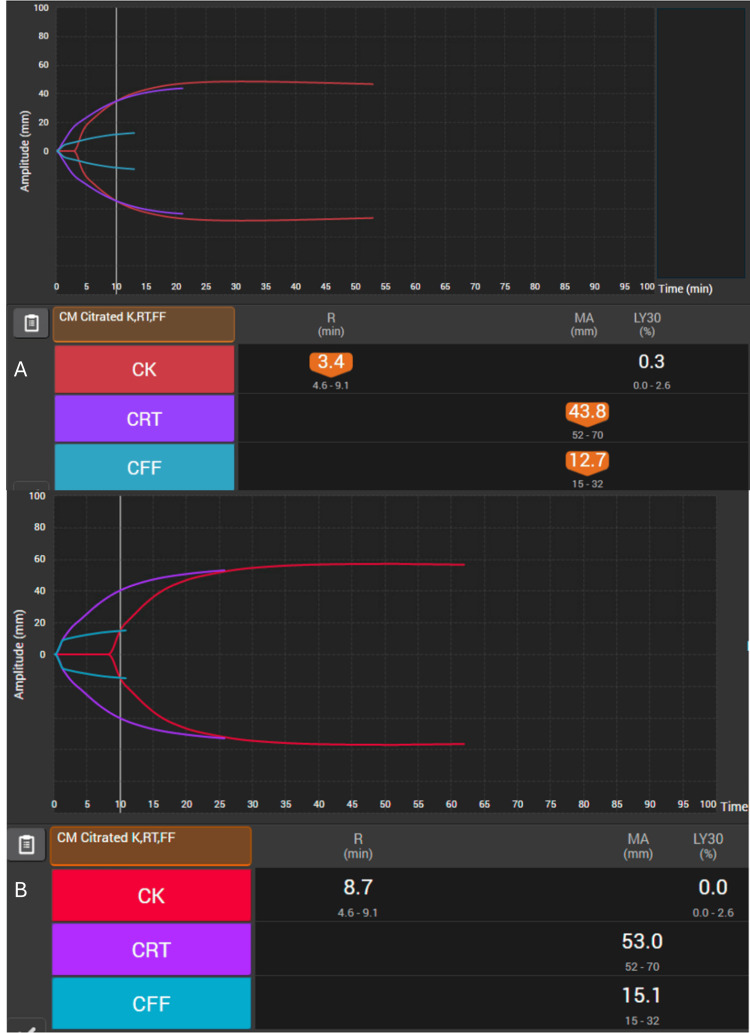
(A) Initial point-of-care TEG with decreased MA CFF, correlated with low fibrinogen performed pre-treatment; (B) Point-of-care TEG demonstrating MA and CFF improvement after fFibrinogen concentrate administration. R: reaction time; MA: maximum amplitude; LY30: lysis at 30 minutes; CK: citrated kaolin; CRT: citrated rapid TEG assay; CFF: functional fibrinogen

The patient was kept overnight on labor and delivery, and the FHR tracing began to show recurrent late decelerations with minimal variability. The decision was made for urgent, repeat cesarean delivery. Combined spinal epidural anesthesia was administered. Cesarean delivery was complicated by PPH with a quantitative blood loss of 1100 mL. Thirty minutes after returning to her room, the patient had continued vaginal bleeding with blood loss now estimated to be ~2000 mL. There was concern for coagulopathy, and repeat labs and TEG^TM^ were obtained (Figure 3). TEG^TM^ showed a decreased maximum amplitude in the functional fibrinogen channel (MA CFF). Traditional labs resulted in a hematocrit of 25.4%, platelet count of 149 K/μL, INR 1.1, and fibrinogen of 156 mg/dL. Tranexamic acid was empirically administered, as were two units of packed red blood cells (pRBCs), and 2g of FC (fibryga®) was administered based upon the pre-testing dosing paradigm in the hospital protocol. Despite transfusion, slow bleeding continued, and repeat labs were largely unchanged except for an INR of 1.3. An additional two units of pRBCs, one unit of FFP, and an additional 70 mg/kg FC (fibryga®) were administered. Repeat labs showed a hematocrit of 26.8% and a fibrinogen of 228 mg/dL. Repeat TEG^TM^ appeared normal. No additional procedures were required, and the patient was discharged home on postoperative day 4.

Trauma

**Figure 4:**
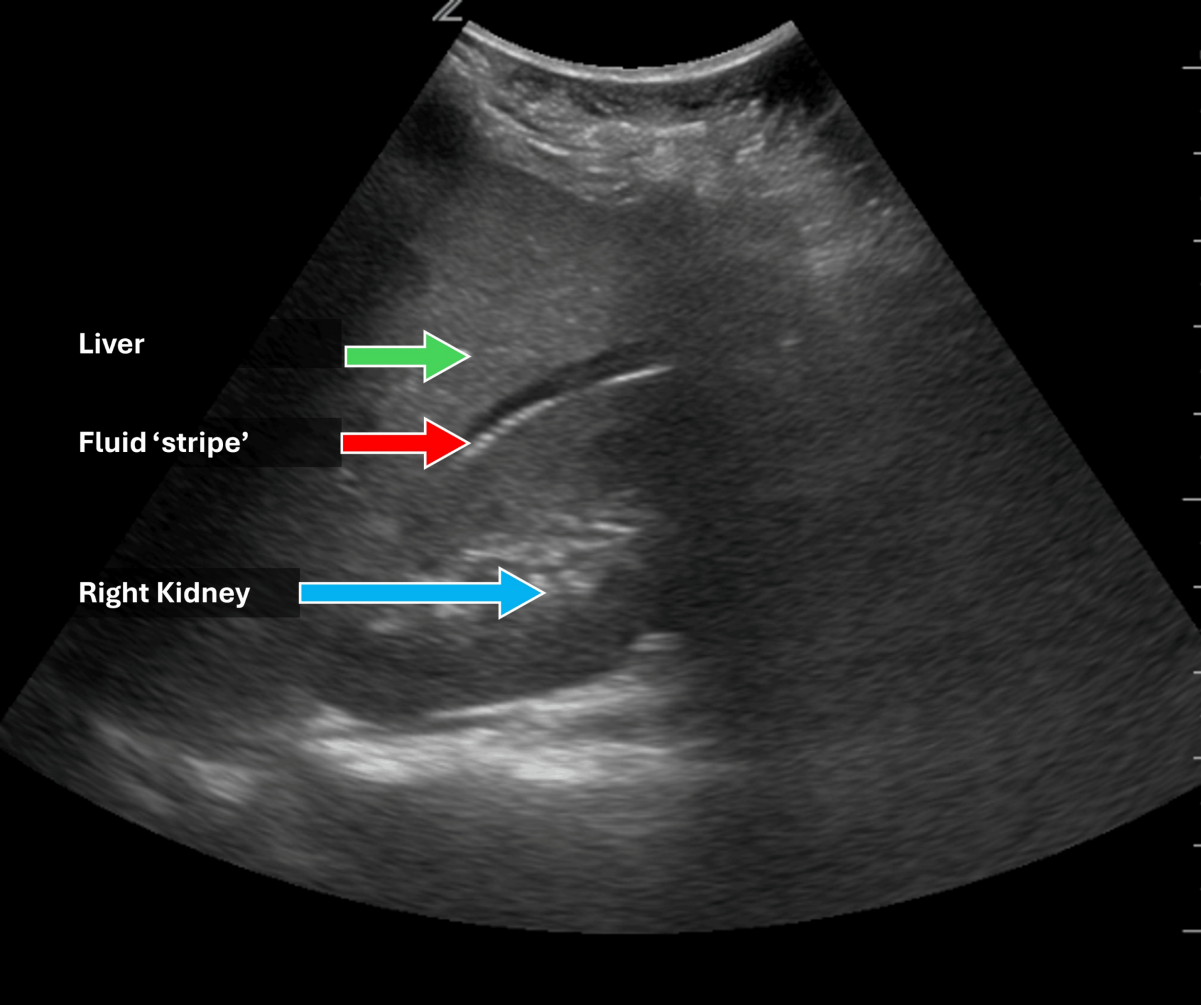
Focused abdominal sonogram for trauma (FAST) exam demonstrating intra-abdominal fluid, suggesting hemorrhage. The FAST exam is performed to examine multiple body cavities: cardiac, right upper quadrant, left upper quadrant, and pelvis to evaluate for free fluid. Here, an anechoic stripe between the right kidney and the inferior surface of the liver suggests intra-abdominal hemorrhage in the setting of hypotension after trauma.

A 57-year-old male was transferred from a rural hospital emergency department following a motor vehicle collision. At presentation, hypotension (blood pressure 82/40 mmHg) and tachycardia (heart rate 133 bpm) were noted. A focused abdominal sonogram for trauma (FAST) exam was positive, prompting immediate transfer to a level 1 trauma center for definitive care (Figure 4).

**Figure 5:**
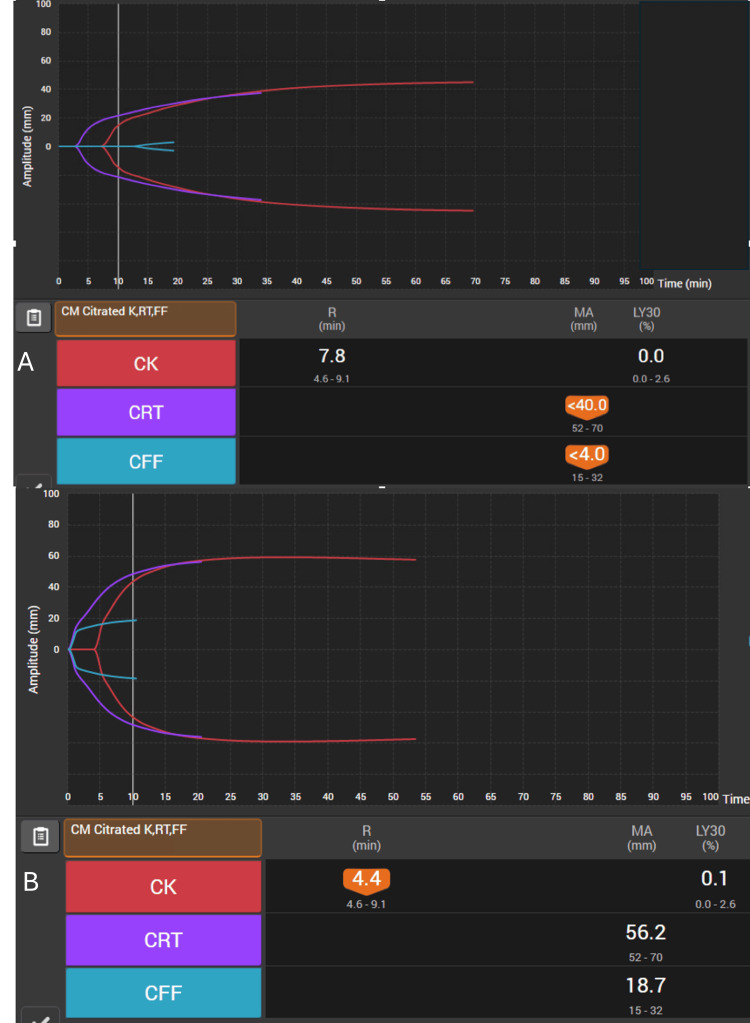
(A) Point-of-care TEG demonstrating reduced MA CFF noted in TEG prior to transfer to a level 1 trauma center and before treatment with FC; (B) Point-of-care TEG demonstrating normalization of MA CFF following FC administration. R: reaction time; MA: maximum amplitude; LY30: lysis at 30 minutes; CK: citrated kaolin; CRT: citrated rapid TEG assay; CFF: functional fibrinogen; FC: fibrinogen concentrate

The patient was initially started on the rural hospital’s massive transfusion protocol and received eight units of pRBCs, eight units of FFP, and a single apheresis platelet unit with a focus on a 1:1:1 hemostatic resuscitation strategy. Despite ongoing resuscitation efforts, pre-transport TEG^TM^ revealed severe AFD, with MA CFF markedly reduced below the normal threshold of 15 mm, indicating significant fibrinogen depletion (Figure 5). Pathogen-reduced cryoprecipitate fibrinogen complex (INTERCEPT®, Cerus Corporation, Concord, CA, USA) was prepared while transfer was being arranged, and 3 grams were administered as the air medical team arrived.

On arrival at the tertiary care hospital 20 minutes later, vital signs were improved (110/45 mmHg and 124 bpm), and he was tachypneic and hypoxic with ongoing tachycardia, and was prepared for the operating room. Repeat TEG^TM^ performed at the start of the procedure indicated that fibrinogen replacement led to the normalization of all hemostatic TEG^TM^ parameters.

Reference ranges for all TEG testing are reported from a variety of resources and are based upon the manufacturer's recommendations [12].

## Discussion

The 1:1:1 resuscitation strategy is important when patient-specific results remain unknown, but even with this paradigm, patients develop hypofibrinogenemia and other disorders contributing to bleeding. Recent analysis suggests fibrinogen repletion is valued at published thresholds, but not beyond; cases presented here provide functional indications via viscoelastic testing in addition to threshold fibrinogen levels. This suggests a role remains for fibrinogen repletion by using FC in AFD [13]. Within the presented cases, several approaches are used for massive hemorrhage management, including fibrinogen repletion.

Cryoprecipitate, a preparation of plasma first developed for hemophilia A in the 1950s, typically contains significantly higher concentrations of fibrinogen, factor VIII, factor XIII, and von Willebrand factor than FFP (Table 2) [11].

**Table 2:** Comparison of fibrinogen to preparations of cryoprecipitate. VWF: von Willebrand factor; TACO: transfusion-associated circulatory overload; TRALI: transfusion-related acute lung injury; IFU: instructions for use * includes transfer/filter device; ** does not include transfer/filter device; *** investment variable Adapted from reference [11].

Characteristic	Fibrinogen Concentrate (fibryga® Fibrinogen, Octapharma, Paramus, NJ, USA)	Fibrinogen Concentrate (Riastap®, CSL Bering, CSL Behring, King of Prussia, PA, USA)	Pathogen Reduced Fibrinogen Complex (INTERCEPT® Fibrinogen Complex, Cerus Corporation, Concord, CA, USA)	Cryoprecipitate
Fibrinogen content per dose	1.0 g/50 mL after reconstitution	0.9-1.3 g/50 mL after reconstitution	Fibrinogen 1556 +/- 248 mg per prepared bag	Variable content of 120-796 mg per 10-20 mL; Dose may be 5 or 10 pooled units based on institution norms
Constituent components	Fibrinogen and fibrin-stabilizing factor (factor XIII)	Fibrinogen, albumin	Fibrinogen, factor VIII, factor XIII antigen, von Willebrand factor antigen	Fibrinogen, factor VIII, factor XIII, VWF, fibronectin
Acceptable storage duration	48 months	60 months	12 months	12 months
Storage requirements	25°C (room temperature)	2-8°C (refrigerated)	Per cryoprecipitate standard pre-treatment; 20-24°C (after preparation)	-18°C (frozen)
Preparation process	Reconstitution with sterile water	Reconstitution with sterile water	Thawing and proprietary process	Thawing
Time to prepare (minutes)	5-10 minutes (reconstitution)	5-10 minutes (reconstitution)	15-20 min (after cryoprecipitate thawed)	20-40 min (thaw)
Stability after preparation	4 hours at room temperature	8 hours at room temperature	5 days after preparation, immediately available for use	4-6 hours or stored refrigerated for 24 hours
Viral mitigation	Inactivation and removal via solvent detergent, nanofiltration	Inactivation and removal via glycine precipitation, heat pasteurization, and cryoprecipitation	Ultraviolet light + amotosalen	Donor screening and source plasma testing
Burden of volume per vial	50 mL	50 mL	Pooled volume ~100 mL	Pooled volume ~100 mL
Cross-matching/ABO	No cross-matching required	No cross-matching required	Requires ABO compatibility	Requires ABO compatibility
Instructions for use (dosing)	Per IFU/product insert	Per IFU/Product insert	Per protocol/institutional standard	Per protocol/institutional standard
Indicated for acquired fibrinogen deficiency	Yes	No	Yes	Yes
Cost	US$900 (wholesale price per single vial via drugs.com)*	US$1550 (wholesale price per single vial via drugs.com)**	Cryoprecipitate cost plus technology investment***	$150-650/unit
Risks	Very low; negligible risk of alloimmunization, TACO, TRALI	Very low; negligible risk of alloimmunization, TACO, TRALI	Low risk of alloimmunization, higher risk of TACO, TRALI	Low risk of alloimmunization, higher risk of TACO, TRALI

The CRYOSTAT-2 clinical trial, investigating early repletion with cryoprecipitate versus standard transfusion protocols in trauma, did not show an improvement in 28-day mortality with the use of cryoprecipitate; however, the authors point out that the median time to first cryoprecipitate transfusion was more than an hour after patient arrival due to the logistical challenges of preparing cryoprecipitate, particularly thaw time [14,15]. Additional challenges with cryoprecipitate include its derivation from multiple blood donors, resulting in variability of fibrinogen per dose, and wastage if unused. Risk of viral transmission remains a factor, spurring development of alternative treatments for hemophilia, and the reason it is not typically used in the European Union (EU) for hypofibrinogenemia [4].

In 2020, the FDA approved a blood system for producing pathogen-reduced cryoprecipitate (INTERCEPT®) for the treatment and control of bleeding in the setting of fibrinogen deficiency [16]. The system’s pathogen reduction process reduces the risk of viral transmission and extends shelf stability to five days after thawing. When stored thawed, the product can be immediately available, but without sufficient utilization, as that thawed storage duration can result in wastage.

Distinctly, FC offers advantages over pathogen-reduced cryoprecipitate, particularly in the context of rapid fibrinogen repletion during major hemorrhage. It allows for immediate reconstitution, precise dosing, and a lower risk of transfusion-related complications due to its viral inactivation process. These factors make FC a highly efficient option for targeted fibrinogen replacement, especially in time-sensitive surgical and trauma settings.

Pathogen-reduced cryoprecipitate, however, may offer additional hemostatic benefits by containing factor VIII, factor XIII, and von Willebrand factor, which may be advantageous in conditions requiring broader coagulation support, such as perioperative bleeding in patients with congenital or other acquired factor deficiencies. Pathogen-reduced cryoprecipitate has a somewhat extended post-thaw shelf life of up to five days, so when kept reconstituted for readiness, there is a risk of wastage.

An Austrian trial investigated fibrinogen repletion with FC, randomizing 100 trauma patients with coagulopathy (identified by VET) to treatment with FFP or FC [17]. This study found a reduced risk of rescue therapy, massive transfusion, and multiple organ failure in the FC group. In the FIBRES trial, FC demonstrated non-inferiority to cryoprecipitate in cardiac surgery [13,15]. Specifically, post-bypass transfusion requirements were equal to treatment with cryoprecipitate. Due to its speed of preparation, viral inactivation, and lower likelihood of wastage, it may provide several practical advantages over cryoprecipitate [4]. In 2024, the FDA approved FC (fibryga®), a virus-inactivated lyophilized powder that is shelf stable and can be rapidly reconstituted in emergency bleeding settings for the treatment of AFD. RiaSTAP® (CSL Behring, King of Prussia, PA, USA) is a cryoprecipitate-derived FC, similar in indication, but requires refrigeration for storage prior to reconstitution.

Recent clinical evidence does demonstrate advantages to the use of FCs in obstetric hemorrhage [18], cardiac surgery-associated hemorrhage [19], and in trauma resuscitation [20], suggesting the inclusion of FC in hospital protocols.

The decision regarding which product to use depends on local hospital practice and logistics. At high-volume centers where fibrinogen replacement is frequently needed, pathogen-reduced cryoprecipitate may be a suitable option due to its predictable utilization and on-demand availability. Conversely, in lower-volume or less predictable settings, FC may offer advantages due to its extended shelf stability, reduced wastage, and precise dosing, making it a more practical choice for managing fibrinogen deficiency in significant bleeding.

Guidelines suggest fibrinogen monitoring in major bleeding [10]. The most common fibrinogen laboratory test, the Clauss assay, takes over an hour to generate results and thus is not relevant in emergent bleeding scenarios. VET, such as TEG^TM^ or rotational thromboelastometry (ROTEM^TM^, Werfen, Bedford, MA, USA), is also recommended, but due to the lack of widespread availability, or in cases of extreme bleeding, clinicians may have to proceed empirically [10]. MA CFF, for example, has been shown to correlate well with the Clauss assay, with an MA CFF of 12 mm corresponding to a fibrinogen level of 150 mg/dL, a threshold frequently cited in multiple guidelines as requiring correction in hemorrhagic settings. The MA CFF result is usually available in 10 minutes or less, allowing action earlier than the Clauss assay. This underscores the utility of point-of-care VET in detecting fibrinogen deficiency and guiding targeted replacement. Furthermore, it may help explain the failure of several studies on fibrinogen therapy, where correction was performed empirically rather than based on real-time coagulation assessment.

## Conclusions

For dynamic clinical scenarios such as intraoperative, obstetrical, traumatic, or other hemorrhage, institutions should continually re-evaluate their protocols. The availability of a system to ready pathogen-reduced cryoprecipitate and the commercial availability of virus-inactivated FC as rapidly reconstituted pharmaceutical products for AFD assures clinicians have opportunities for immediate treatment. Each should be considered in massive transfusion and hemorrhage control protocols, with special attention to the needs of each institution. Despite advancements in fibrinogen replacement strategies, several critical questions remain unanswered. The optimal fibrinogen threshold for correction is still debated, and it is unclear whether target levels should vary across different bleeding scenarios, such as various surgical settings. Furthermore, the growing reliance on point-of-care VET suggests that fibrinogen replacement should be guided by objective hemostatic parameters rather than empiric administration alone. Targeting supraphysiologic fibrinogen levels without clear evidence may not only be unnecessary but could also pose potential risks. Future research should focus on defining precise correction thresholds and refining patient-specific strategies to optimize outcomes while minimizing transfusion-related complications.
